# Tuberculosis disease burden trends, drivers, and projections to 2050 in China and the wider Central Asian region, 1990–2023

**DOI:** 10.1371/journal.pone.0358349

**Published:** 2026-10-09

**Authors:** Bahetibieke Tuohetaerbaike, Dina Mulali, Mehdi Borhani, Kai Wang, Wenbao Zhang, Hao Wen

**Affiliations:** 1 State Key Laboratory of Pathogenesis, Prevention and Treatment of High Incidence Diseases in Central Asia, Xinjiang Medical University, Urumqi, China; 2 Respiratory and Critical Care Medicine Center, People’s Hospital of Xinjiang Uygur Autonomous Region, Urumqi, Xinjiang, China; 3 Joint Key Laboratory for International Cooperation on Major Diseases in Central Asia, Xinjiang Medical University, Urumqi, China; 4 College of Medical Engineering and Technology, Xinjiang Medical University, Urumqi, Xinjiang, China; 5 Institute of Medical Engineering Interdisciplinary Research, Urumqi, Xinjiang, China; 6 Clinical Medicine Research Institute, The First Affiliated Hospital of Xinjiang Medical University, Urumqi, Xinjiang, China; 7 Department of Hepatobiliary and Hydatid Surgery, The First Affiliated Hospital of Xinjiang Medical University, Urumqi, China; National Center for Chronic and Noncommunicable Disease Control and Prevention, Chinese Center for Disease Control and Prevention, CHINA

## Abstract

**Background:**

Tuberculosis (TB) remains a leading infectious killer, with high burden concentrated in China and wider Central Asia. Integrated long-term trend analysis and forecasting for this region are scarce. We aimed to quantify TB burden trends, drivers, inequalities, and 2050 projections across China and wider Central Asia (1990–2023).

**Methods:**

We used Global Burden of Disease 2023 data to estimate age-standardized incidence, prevalence, mortality, and disability-adjusted life years (DALYs). Joinpoint, age-period-cohort, decomposition, and Bayesian APC models were applied. Socioeconomic inequalities were assessed via Socio-demographic Index (SDI) and Concentration Index.

**Results:**

China achieved steep declines in age-standardized mortality (−94.2%) and DALYs (−92.4%), driven by epidemiological gains offsetting population growth and aging. Central Asia improved moderately but heterogeneously; population growth diluted gains, with Tajikistan showing rising absolute burden. TB burden correlated negatively with SDI; socioeconomic inequality fell markedly, especially for mortality (−69.0%), but persisted among low-SDI groups. By 2050, model projections suggest that mortality targets may be achievable under current trends, although uncertainty remains substantial.

**Conclusions:**

China has undergone a substantial TB epidemiological transition characterized by marked declines in mortality and DALYs. Targeted interventions-latent TB treatment in China, accelerated case finding and cross-border collaboration in Central Asia-and strengthened regional cooperation are critical to advance regional TB elimination.

## 1. Introduction

Tuberculosis was estimated by the World Health Organization (WHO) to be the world’s leading cause of death attributable to a single infectious agent in 2023, responsible for approximately 1.25 million fatalities nearly twice the mortality burden of HIV/AIDS and exceeding that of malaria. Despite decades of global control efforts, progress toward TB elimination remains slow and uneven, especially in regions affected by epidemiological transition, demographic expansion, and fragile health systems [1]. TB is fundamentally a disease of poverty, social determinants such as undernutrition, overcrowded housing, and limited healthcare access contribute substantially to global incidence [2,3]. In 2023, these structural drivers translated into 10.8 million incident cases worldwide, with multidrug-resistant or rifampicin-resistant TB (MDR/RR-TB) accounting for an estimated 400,000 cases, only 44% of which were diagnosed [1]. Worldwide annual TB deaths declined steadily from 2005 to 2019, reaching 1.4 million in that year. However, this downward trend reversed in 2020, when deaths rose to 1.5 million, and by 2021, global TB mortality had returned to the level recorded in 2017 [4]. These challenges are nowhere more acute than in the geographically contiguous region of China and wider Central Asia. While this zone accounts for a significant portion of the global population, its TB burden is disproportionately concentrated. China alone comprises 9% of global cases, and Central Asia reports over 34,000 cases annually with incidence rates up to 112 per 100,000- significantly higher than the average in Europe [5].

MDR/RR-TB rates are particularly concerning; in Kazakhstan, 26% of new cases are MDR-TB-far exceeding the global average of 3.2% and four of the five Central Asian countries are classified among the world’s high MDR-TB burden countries [5–8]. During the COVID-19 pandemic, diagnostic capacity was severely disrupted across Central Asia and the Caucasus. Funding for TB control and prevention programs remains precarious, with limited domestic financing, unsustainable health aid models, and weak multi-sectoral collaboration-all of which constrain progress toward ending TB in this high-burden region [5,8–13].

China, home to approximately 18% of the global population and historically one of the world’s highest TB-burden countries, has made substantial progress through sustained political commitment and targeted national strategies for tuberculosis control and prevention [14,15]. Key nationwide interventions included universal free testing and first-line drug treatment for drug-susceptible tuberculosis; subsidized second-line MDR/RR-TB treatment packages with partial government reimbursement for retreatment failure patients, alongside universal Bacillus Calmette-Guérin (BCG) vaccination and a robust grassroots surveillance network [16–19]. Despite these gains, China still faces substantial absolute burden, with an estimated 748,000 new TB cases annually (2022 data), a large reservoir of latent TB infection (350 million), and persistent MDR-TB challenges [1,20]. Population aging and urban-rural health inequalities remain critical concerns, with TB burden concentrated in low-socioeconomic status populations and elderly groups [21].

Despite growing attention to regional TB dynamics, critical knowledge gaps persist. Existing literature largely consists of localized or country-specific studies, often limited to short time periods or single burden indicators. For instance, Sun et al. (2025) conducted a comprehensive analysis of China’s TB burden from 1990 to 2021 using Global Burden of Disease Database (GBD), examining age-standardized rates and age-period-cohort effects, but focused solely on China without regional comparison [22]. Similarly, Kussainova et al. (2026) analyzed social determinants of TB incidence across the five Central Asian countries (2000–2023) and forecasted trends to 2030, yet did not decompose burden drivers or assess health inequalities using SDI and concentration indices [10]. A systematic review protocol by Idayat et al. (2025) aims to synthesize TB prevalence data across Central Asia and the Southern Caucasus, strengthening surveillance, improving data collection, and conducting longitudinal studies are essential for designing effective TB control strategies in these regions [5]. While a global study by Jiang et al. (2026) applied decomposition analysis, health inequality assessment, and frontier analysis to TB-related DALYs across 204 countries and territories, it did not provide integrated, long-term forecasts specifically for the China-Central Asia region [23].

With the End TB Strategy’s 2030 target deadline approaching and the China-Central Asia economic corridor poised to accelerate cross-border population movement, alongside nearly 380 million internal migrants in China alone [24], the absence of integrated regional evidence undermines both national planning and regional cooperation. Current forecasts for Central Asia reveal highly heterogeneous trends among countries, with some showing non-significant declines, highlighting the need for better integration of population dynamics into predictive models [10], Concurrently, China’s aging population is shifting the tuberculosis burden disproportionately toward elderly cohorts [25,26], while cross-border transmission risks, particularly along the rapidly developing economic corridors, present an additional challenge that current elimination strategies have yet to fully address [27].

To address these critical knowledge gaps, the present study sought to delineate long-term temporal trends in TB incidence, prevalence, mortality, and disability-adjusted life years across China and the wider Central Asian region from 1990 to 2023, with stratification by age, sex, and country. We further aimed to decompose changes in the absolute disease burden into the separate contributions of population growth, population aging, and epidemiological change, and to quantify socioeconomic inequalities using the Socio-demographic Index (SDI) and Concentration Index. We also projected future trajectories of tuberculosis burden through 2050 using Bayesian age-period-cohort models to evaluate progress toward the World Health Organization End TB Strategy targets. By integrating high-quality data from the Global Burden of Disease 2023 study and applying rigorous statistical methods, this analysis provides a comprehensive evidence base to support the optimization of regional tuberculosis control policies, strengthen cross-border collaboration between China and Central Asia, and advance global efforts toward tuberculosis elimination.

## 2. Methods

### 2.1 Study setting and data sources

This ecological time-series study leverages datasets from the GBD, Injuries, and Risk Factors Study 2023, the most comprehensive and contemporaneous global health burden assessment to date. GBD 2023 generates systematic, internally consistent estimates of incidence, prevalence, mortality, and disability-adjusted life years (DALYs) covering 375 diseases and injuries across 204 countries and territories, with stratified outputs by age, sex, and calendar year spanning 1990–2023 [28]. All underlying GBD datasets are freely accessible via the Global Health Data Exchange (GHDx) online query platform (https://vizhub.healthdata.org/gbd-results/).

For the estimation of disease incidence, prevalence and DALYs in GBD 2023, heterogeneous primary data streams including vital registration, disease notification registries, population-representative household surveys, routine public health surveillance datasets, and published peer-reviewed epidemiological literature-were pooled via the standardized Bayesian meta-regression tool DisMod-MR 2.1 [28]. This framework enforces unified data cleaning and harmonization, incorporating outlier elimination, multiple imputation, cross-country alignment of case definitions, and quantitative correction for underdiagnosis biases in low-resource settings. All analytical datasets used in this work were downloaded directly from the GHDx public repository.

Within the GBD framework, all age‑standardized burden metrics are generated with 95% uncertainty intervals (UIs), whose width inherently reflects the quantity and consistency of underlying input data. We leverage this native uncertainty characterization to qualitatively assess cross‑national disparities in surveillance infrastructure robustness—with broader UIs indicating sparser registration coverage, as typically observed for data‑scarce settings such as Tajikistan and Turkmenistan.

On the GBD website, TB is categorized into distinct states、 including latent tuberculosis infection, drug-susceptible tuberculosis, multidrug-resistant tuberculosis (MDR-TB) without extensive drug resistance, and extensively drug-resistant tuberculosis. In contrast, the tuberculosis prevalence estimates reported by the WHO conventionally refer specifically to active tuberculosis disease, encompassing both bacteriologically confirmed and clinically diagnosed cases, and generally exclude latent infections. Consequently, when the GBD estimates encompass all TB infection states, its reported prevalence figures are typically substantially higher than those published by the WHO.

### 2.2 Study indicators

For this study, we extracted age-standardized rates (ASRs) and case numbers for four core TB burden indicators incidence, prevalence, mortality, and DALYs, the latter representing the sum of years of life lost (YLLs) and years lived with disability (YLDs). Data were extracted for the period 1990–2023, stratified by location, age (5-year age groups from 0–4–95 + years), sex (male, female, and both), metric (number and rate per 100,000 population), and measure (crude and age-standardized rates).

### 2.3 Study location definition and justification

This study encompasses 10 locations across inner continental Asia. According to the GBD 2023 location hierarchy, the “Central Asia” region comprises nine countries (Armenia, Azerbaijan, Georgia, Kazakhstan, Kyrgyzstan, Mongolia, Tajikistan, Turkmenistan, and Uzbekistan). To this core set, we added China classified under East Asia in the GBD hierarchy-to capture the full spectrum of tuberculosis dynamics across the contiguous landmass of inner Asia, forming a geographically and epidemiologically interconnected study area stretching [28,29]. Throughout the manuscript, we refer to these nine countries collectively as the ‘wider Central Asian region’ for brevity, while acknowledging that this expanded definition includes the Caucasus countries and Mongolia alongside the five Central Asian republics.

While Caucasus nations differ geographically from five Central Asian republics and China, several shared epidemiological and policy rationales support unified regional analysis. First, all three Caucasus states rank among global high MDR-TB burden countries with comparable rifampicin resistance prevalence to Central Asian republics, creating shared cross-border transmission risks within inland Eurasian mobility corridors. Second, all included territories fall under consistent GBD 2023 “Central Asia” regional aggregation frameworks, enabling standardized cross-country comparison without methodological bias. Third, Belt and Road cross-border population flows connect Caucasus, Central Asia and China through shared trade and migrant routes, generating interconnected TB surveillance challenges that demand integrated regional burden assessment.

This grouping is further justified by four complementary considerations. first, GBD regional consistency, as our definition aligns with the GBD “Central Asia” regional aggregate covering Caucasus countries to secure methodological consistency with the underlying data source; second, shared epidemiological characteristics, given that all nine nations carry a substantial MDR-TB burden and undergo continuous demographic shifts that set them apart from other Asian subregions; third, geographic and economic integration, whereby growing cross-border human mobility driven by the Belt and Road Initiative necessitates unified regional coordination for tuberculosis surveillance and intervention, validating the region as a cohesive epidemiological unit for analysis; and fourth, policy relevance, stemming from expanding collaboration among national tuberculosis control programs across these countries via the China-Central Asia Tuberculosis Control Research Base, which generates practical demand for consolidated regional epidemiological evidence.

### 2.4 Statistical analysis

Descriptive analysis was conducted to characterize the TB disease burden across China, the wider Central Asian region, and different SDI regions. We analyzed variations in TB burden across geographic areas, and performed detailed stratification by age groups and sex to comprehensively evaluate the TB disease burden in China and the broader study region.

To quantify temporal trends in age-standardized TB burden rates from 1990 to 2023, Joinpoint regression (version 4.8.0.1) was employed using the segmented regression method [30]. The maximum number of joinpoints was set to 2. Autocorrelation in time-series was adjusted in the Joinpoint model. The Average Annual Percent Change (AAPC) and corresponding 95% confidence interval (CI) were calculated. A positive AAPC indicated an increasing trend, while a negative AAPC indicated a decreasing trend. Statistical significance was determined using the Monte Carlo permutation test, with P < 0.05 considered statistically significant.

The age-period-cohort (APC) model based on the Poisson distribution was applied to simultaneously estimate the descriptive trends of age, period, and birth cohort on TB disease burden [31]. Analysis was performed using a web-based analytical tool developed by the National Cancer Institute of the United States. To meet equal interval requirements for age, period, and cohort, the study period was divided into consecutive 5-year intervals. Model outputs included local drift values, longitudinal age curves, and period or cohort rate ratios (RR) [32–34]. Local drift values represented annual percentage changes in disease rates for specific age groups; longitudinal age curves reflected age-specific rates adjusted for period effects; an RR > 1 indicated elevated relative risk, and an RR < 1 indicated reduced relative risk. The APC model is affected by the identifiability problem (linear dependence between age, period, and cohort). Results should be interpreted as descriptive trends, not causal effects.

Decomposition analysis was performed using the method established by Das Gupta [35,36] to partition the overall changes in TB burden into three distinct components: population aging, population growth, and epidemiological changes. Mathematical decomposition techniques were used to quantify the individual contribution of each factor to the observed changes in disease burden.

Bayesian Age-Period-Cohort (BAPC) models were implemented using integrated nested Laplace approximations (INLA) to estimate posterior marginal distributions [37,38]. Projections were generated using Bayesian models implemented via the INLA package in R software (version 4.4.1). Models were fitted with a weakly informative Gaussian prior on the intercept and a random walk prior (rw2) on the time trend to allow flexible temporal smoothing while preventing overfitting. Model validation was performed using out-of-sample temporal cross-validation; we masked the most recent 10 years of GBD data (2014–2023), fitted BAPC models on 1990–2013 data, then compared predicted versus observed age-standardized incidence, prevalence, mortality, and DALY rates across all study countries. Mean absolute error (MAE) was calculated as the primary measure of forecast accuracy. MAE values from the validation period were also used to construct empirical prediction intervals (2 × MAE) for the 2024–2050 projections (S1 Table). All data from 1990 to 2023 were organized and preprocessed in Microsoft Excel 2021, and all statistical analyses and visualizations were performed using R software.

### 2.5 Ethics statement

This study was conducted using publicly available, de-identified secondary data from the GBD 2023 study. No individual patient-level data were collected or analyzed, and no human participants were directly involved in this research. Therefore, ethical approval and informed consent for participation were not required for this ecological time-series analysis, in accordance with relevant research ethics guidelines.

## 3. Results

### 3.1 Temporal trends in TB burden (1990–2023)

#### 3.1.1 Overall estimated annual percentage change (EAPC).

All age-standardized TB burden indicators decreased significantly in China and all Central Asian countries, but substantial disparities existed across Central Asian nations (Table 1).

**Table 1:** Age-standardized TB burden in China and the wider Central Asian region, 1990 vs 2023, with estimated annual percentage change (EAPC), 1990–2023.

Location	Indicator	1990 ASR (per 100,000)	2023 ASR (per 100,000)	EAPC (95% CI)	% Change
China	Incidence	151.02	40.33	−4.44 (−4.62 to −4.26)	−73.3%
	Prevalence	33,241	31,770	−0.27 (−0.42 to −0.12)	−4.4%
	DALYs	753.96	57.61	−8.08 (−8.27 to −7.88)	−92.4%
	Deaths	22.16	1.29	−8.61 (−8.85 to −8.37)	−94.2%
Central Asia (regional)	Incidence	107.63	51.50	−2.52 (−2.91 to −2.12)	−52.1%
	Prevalence	35,453	18,988	−2.00 (−2.14 to −1.86)	−46.4%
	DALYs	657.38	214.52	−4.87 (−5.80 to −3.92)	−67.4%
	Deaths	13.42	4.54	−4.76 (−5.69 to −3.83)	−66.2%
Kazakhstan	Incidence	122.03	46.90	−3.45 (−4.14 to −2.76)	−61.6%
	Prevalence	33,241	15,709	−2.54 (−2.79 to −2.30)	−52.7%
	DALYs	562.40	92.83	−7.79 (−9.45 to −6.11)	−83.5%
	Deaths	11.28	1.95	−7.70 (−9.30 to −6.06)	−82.7%
Kyrgyzstan	Incidence	110.65	67.84	−1.14 (−1.48 to −0.79)	−38.7%
	Prevalence	35,328	17,567	−2.18 (−2.33 to −2.03)	−50.3%
	DALYs	549.67	150.16	−5.00 (−6.28 to −3.70)	−72.7%
	Deaths	10.45	3.18	−4.78 (−6.09 to −3.44)	−69.6%
Tajikistan	Incidence	155.48	83.13	−2.31 (−2.60 to −2.01)	−46.5%
	Prevalence	37,966	22,455	−1.54 (−1.65 to −1.42)	−40.8%
	DALYs	1,171.6	561.65	−3.73 (−4.34 to −3.11)	−52.1%
	Deaths	22.84	12.73	−3.27 (−3.88 to −2.66)	−44.3%
Turkmenistan	Incidence	85.36	46.82	−2.11 (−2.38 to −1.85)	−45.2%
	Prevalence	36,641	19,484	−1.93 (−2.04 to −1.83)	−46.8%
	DALYs	652.56	342.70	−3.08 (−3.88 to −2.28)	−47.5%
	Deaths	13.01	6.74	−3.19 (−4.00 to −2.36)	−48.2%
Uzbekistan	Incidence	97.68	47.27	−2.18 (−2.48 to −1.87)	−51.6%
	Prevalence	35,037	19,320	−1.89 (−2.01 to −1.76)	−44.9%
	DALYs	523.01	174.82	−4.13 (−5.13 to −3.11)	−66.6%
	Deaths	10.88	3.82	−3.82 (−4.80 to −2.84)	−64.9%
Armenia	Incidence	42.77	15.81	−2.65 (−3.20 to −2.10)	−63.0%
	Prevalence	30,872	14,517	−2.36 (−2.42 to −2.29)	−53.0%
	DALYs	190.30	34.99	−5.81 (−6.82 to −4.78)	−81.6%
	Deaths	3.50	0.66	−5.69 (−6.84 to −4.52)	−81.1%
Azerbaijan	Incidence	89.99	39.00	−3.13 (−3.52 to −2.75)	−56.7%
	Prevalence	35,760	17,705	−2.23 (−2.39 to −2.07)	−50.5%
	DALYs	729.77	255.29	−4.90 (−5.50 to −4.29)	−65.0%
	Deaths	14.31	4.60	−5.09 (−5.71 to −4.47)	−67.9%
Georgia	Incidence	59.75	40.08	−2.68 (−5.98 to 0.75)	−32.9%
	Prevalence	29,354	16,458	−1.07 (−1.83 to −0.30)	−43.9%
	DALYs	24.98	7.08	−0.76 (−0.80 to −0.30)	−76.0%
	Deaths	0.99	0.21	−0.76 (−0.81 to −0.71)	−75.8%
Mongolia	Incidence	219.36	55.96	−4.56 (−4.95 to −4.16)	−74.5%
	Prevalence	42,371	20,550	−2.36 (−2.49 to −2.22)	−51.5%
	DALYs	3,050.9	476.83	−6.28 (−6.58 to −5.97)	−84.4%
	Deaths	67.09	10.21	−6.39 (−6.70 to −6.07)	−84.8%

China exhibited the most rapid decline in all indicators; age-standardized DALY rate (ASDR) fell from 753.96 to 57.61 per 100,000 (EAPC = −8.08, 95% CI: −8.27 to −7.88), and age-standardized mortality rate (ASMR) declined with an EAPC of −8.61 (95% CI: −8.85 to −8.37).

Among Central Asian countries, Kazakhstan and Armenia achieved the most rapid burden reduction (ASDR EAPC: −7.79 and −5.81, respectively), while Turkmenistan and Tajikistan experienced the slowest declines (ASDR EAPC: −3.08 and −3.73, respectively). Central Asia’s regional average ASDR EAPC (−4.87) was 39.7% slower than China’s.

Across all locations, mortality (ASMR) declined fastest (regional average EAPC = −6.68), while prevalence (ASPR) declined slowest (regional average EAPC = −1.85), with China’s ASPR showing the weakest downward trend (EAPC = −0.27).

TB burden declined sharply across all locations, but Central Asian countries retained substantially higher absolute burden than China in 2023 (Fig 1).

**Fig 1:**
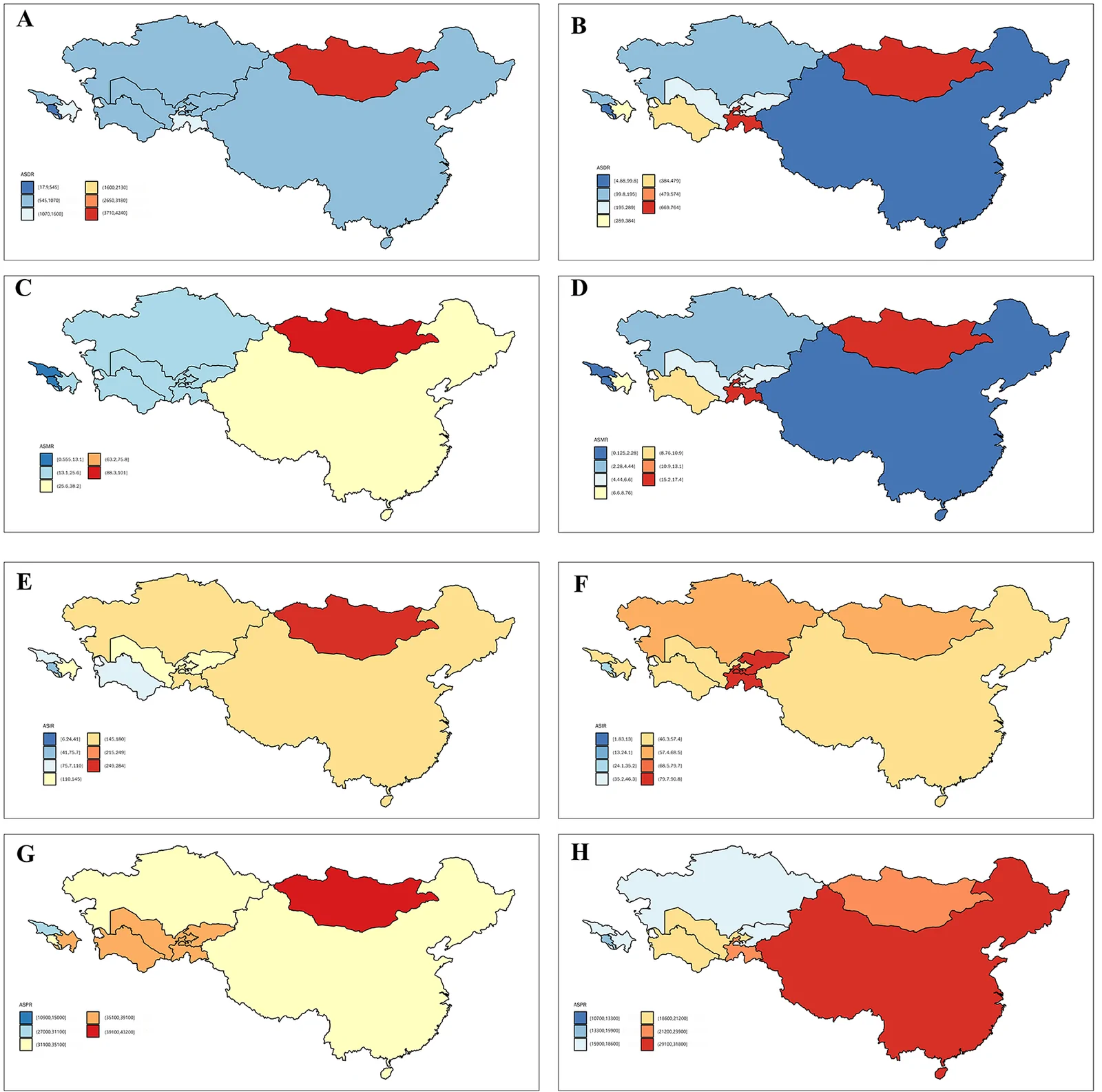
Age-standardized TB burden in China and the wider Central Asian region, 1990 vs 2023. (A) ASDR; (B) ASMR; (C) ASIR; (D) ASPR. Error bars represent 95% uncertainty intervals. Base map layers were derived from the public domain Natural Earth dataset, and all TB burden estimates were sourced from the GBD 2023 study, available from the GHDx. The figure was entirely generated by the authors using R software.

Tajikistan had the highest ASDR in 2023 (561.65 per 100,000), 9.8 times higher than China (57.61 per 100,000) and 6.0 times higher than Kazakhstan (92.83 per 100,000). Turkmenistan’s ASDR (342.70 per 100,000) declined only 47.5% compared to the regional average decline of 67.4%.

China’s ASMR dropped 94.2% (1990: 22.16 to 2023: 1.29 per 100,000), while Tajikistan’s ASMR decreased by 44.3% (1990: 22.84 to 2023: 12.73 per 100,000). Kazakhstan’s ASMR (1.95 per 100,000 in 2023) was the lowest among Central Asian countries, approaching China’s level.

Kyrgyzstan had the slowest ASIR decline (38.7%, 1990: 110.65 to 2023: 67.84 per 100,000), whereas China’s ASIR fell by 73.3% (1990: 151.02 to 2023: 40.33 per 100,000). Azerbaijan’s ASIR decline (56.7%) exceeded the Central Asian average (52.1%).

China’s ASPR shows minimal reduction (4.4%, 1990: 33,241–2023: 31,770 per 100,000), whereas Kazakhstan’s ASPR fell 52.7% (1990: 33,241–2023: 15,709 per 100,000).

#### 3.1.2 Age-sex stratified trends.

Males consistently exhibited higher TB burden than females across all indicators and countries, but the magnitude of disparity varied substantially (Fig 2). China achieved the largest reduction in gender gap, where the male-to-female ASMR ratio decreased from 1.8:1 in 1990 to 1.3:1 in 2023, the steepest decline among all locations. Among Central Asian countries, Georgia had the widest gender gap (male-to-female ASDR ratio = 1.7:1 in 2023), while Armenia had the narrowest (1.4:1). Tajikistan and Turkmenistan showed statistically significant slow decline (EAPC = −0.27, 95% CI −0.42 to −0.12) in gender disparity over the 34-year period, with ratios remaining stable at approximately 1.5:1. This persistent young-adult TB burden in Tajikistan also contributes to the high uncertainty of its future burden projections shown in Section 3.4.

**Fig 2:**
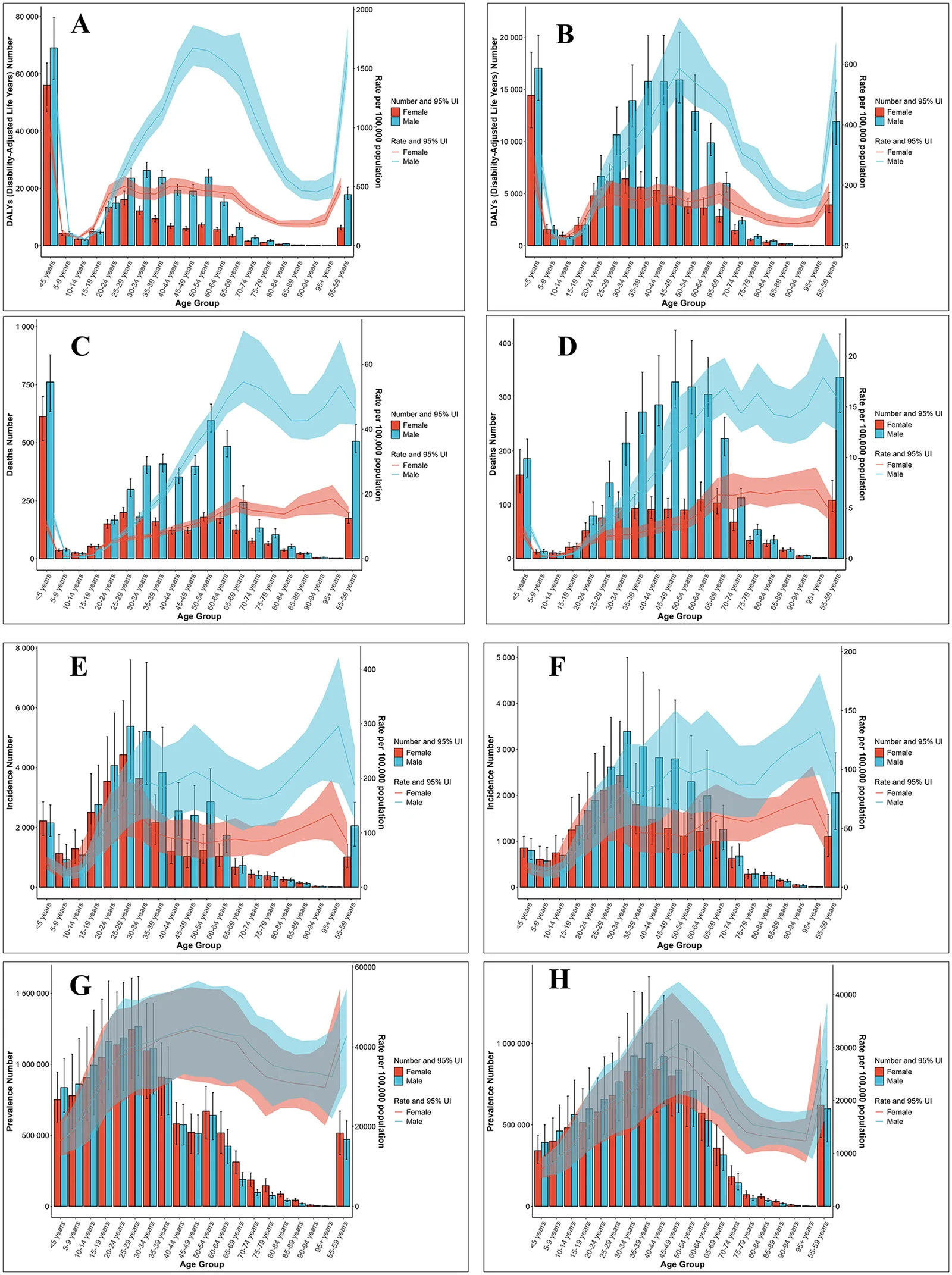
Age-sex specific ASDR of TB in China and the wider Central Asian region, 1990 (A) and 2023 (B).

The age distribution of TB burden shifted dramatically from children toward older adults across all locations, but with marked country-specific heterogeneity (Fig 2). In 1990, children <5 years bore the highest ASDR in China (165.6 per 100,000) and Tajikistan (142.3 per 100,000), representing 22–25% of total DALYs, a pattern reflecting high transmission rates and limited pediatric prevention in the early epidemic. By 2023, China’s burden shifted to adults 25–54 years (ASDR = 28.7 per 100,000), while ASDR in children <5 years plummeted by 95.7% to 7.2 per 100,000, reflecting successful transmission interruption and universal BCG vaccination. In Central Asia, two distinct aging trajectories emerged: Kazakhstan and Armenia transitioned to older adult burden (≥70 years: ASDR = 18.9 and 16.3 per 100,000). Tajikistan retained high burden among adolescents and young adults (10–24 years; ASDR = 32.1 per 100,000), suggesting ongoing community transmission and delayed epidemiological transition.

#### 3.1.3 Inter-regional comparative TB burden disparities.

Stratified inter-regional comparison identified three distinct TB burden archetypes across the full study domain: (1) China, characterized by near-eliminated mortality, slowly plateauing incidence; (2) intermediate-burden countries (Kazakhstan, Armenia, Georgia) with steep historical burden declines converging toward low mortality benchmarks; (3) High-burden countries with strong demographic pressure (Tajikistan, Turkmenistan) with slow per-capita declines and rising absolute case counts driven by population expansion.

### 3.2 Decomposition analysis

We decomposed change in TB burden (1990–2023) into population growth, aging, and epidemiological change. Positive values indicate burden-increasing effects; negative values indicate burden reduction (Fig 3 and Table 2).

**Fig 3:**
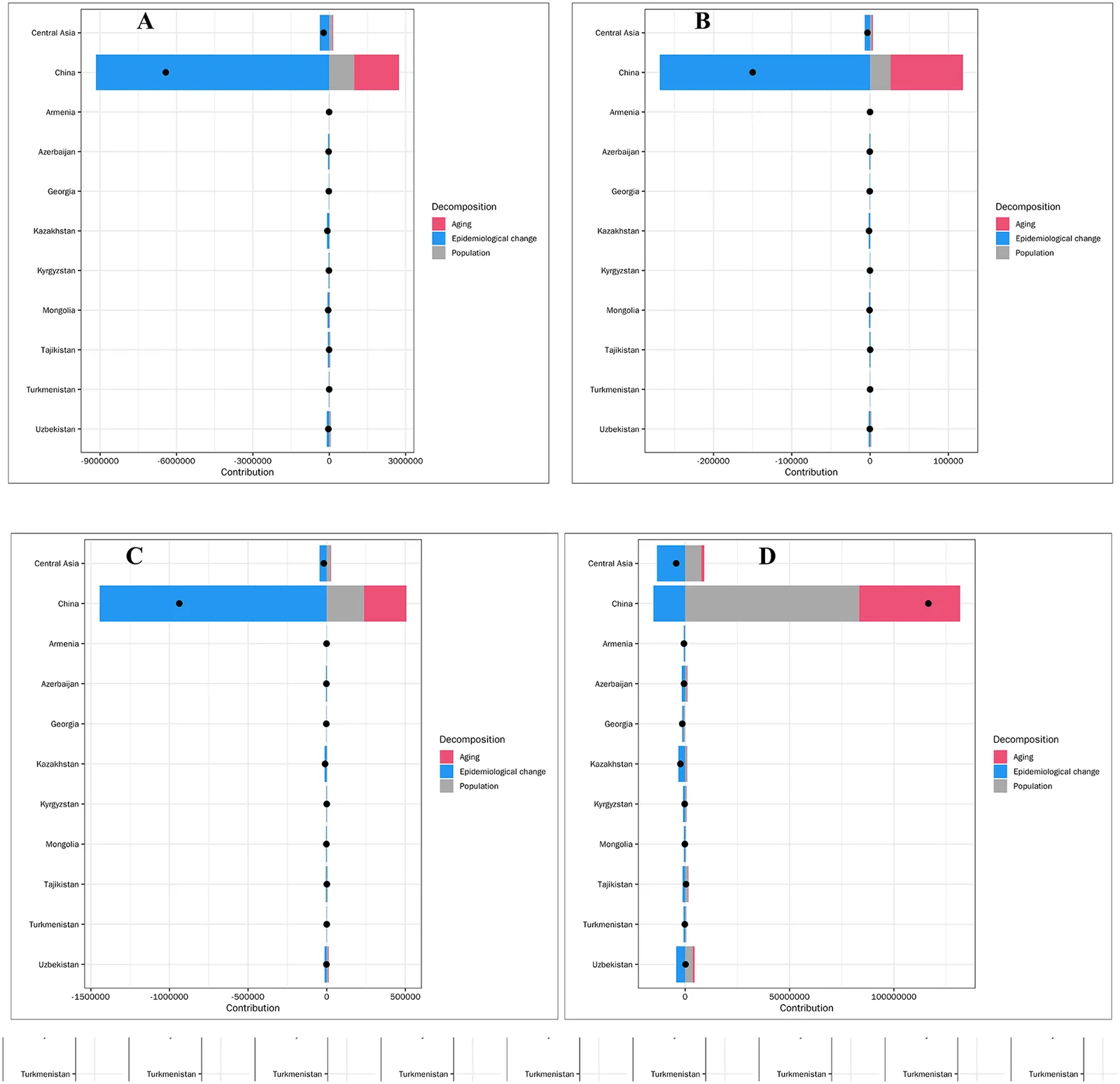
Decomposition of DALY, Deaths, Incidence, and Prevalence change (1990–2023) into population growth, aging, and epidemiological changes in China and wider Central Asian region. Positive values = increased burden; negative values = decreased burden.

**Table 2:** Decomposition of absolute TB burden change, China vs Central Asia, 1990–2023.

Indicator	Region	Overall change	Epidemiological change	Aging	Population
DALYs (×1000)	China	−6,421	−9,163	+1,765	+976
	C. Asia	−220	−371	+25	+126
Deaths	China	−149,968	−268,550	+92,427	+26,156
	C. Asia	−3,347	−6,780	+1,107	+2,326
Incidence	China	−937,434	−1,443,901	+269,532	+236,936
	C. Asia	−18,285	−45,453	+5,164	+22,003
Prevalence (×1000)	China	+116,500	−15,206	+48,340	+83,365
	C. Asia	−4,308	−13,474	+1,460	+7,706

Epidemiological change was the dominant driver of burden reduction across all locations, but its magnitude differed sharply between China and Central Asia (Fig 3A). In China, epidemiological changes alone accounted for a reduction of 9.16 million DALYs, fully offsetting population growth (+0.98 million) and aging (+1.77 million). In Central Asia, Kazakhstan achieved the largest epidemiological DALY reduction (−72,407), followed by Uzbekistan (−34,894) and Kyrgyzstan (−13,251). Tajikistan and Turkmenistan recorded more modest reductions (−6,709 and −3,613, respectively). These magnitudes reflect substantial heterogeneity in the scale of TB control efforts across the region.

TB mortality decomposition mirrored DALY patterns, albeit at smaller absolute magnitudes (Fig 3B). In China, epidemiological changes averted −268,550 deaths, completely offsetting the combined pressure of population growth (+26,156) and aging (+92,427). Among Central Asian countries, epidemiological mortality reductions ranged from −1,708 in Kazakhstan to −16 in Turkmenistan, with intermediate values of −772 (Tajikistan), −411 (Azerbaijan), −398 (Uzbekistan), −362 (Georgia), −190 (Kyrgyzstan), and −87 (Armenia). The relative contribution of epidemiological change to mortality reduction was larger than for DALYs in all locations, reflecting the mortality-focused impact of DOTS and first-line anti-TB treatment scale-up.

Incidence decomposition revealed intermediate epidemiological contributions**,** with China again demonstrating the largest absolute reduction (Fig 3C). Epidemiological changes averted −1.44 million incident cases in China, which was sufficient to offset the combined burden from population growth (+0.24 million) and aging (+0.27 million).

Across Central Asia, the relationship between epidemiological gains and demographic pressures was less consistent. While Kazakhstan, Uzbekistan, and Turkmenistan achieved net incidence declines (overall differences of −10,491, −1,760, and −65, respectively), Kyrgyzstan (+160) and Tajikistan (+820) experienced net increases in absolute incident cases-despite positive epidemiological changes in both countries (−2,443 and −5,592, respectively). These increases were driven by rapid population growth (Kyrgyzstan: + 2,263; Tajikistan: + 5,658), highlighting a fundamental challenge for TB elimination in high-fertility settings.

Prevalence decomposition revealed a fundamentally different and ostensibly paradoxical pattern (Fig 3D). Unlike DALYs, mortality, and incidence, population growth and aging dominated changes in prevalence, whereas epidemiological gains exerted only a weak mitigating effect.

In China, population growth (+83.4 million prevalent cases) and aging (+48.3 million) together added +131.7 million prevalent cases over 1990–2023. Epidemiological changes reduced prevalence by only −15.2 million cases-offsetting just 11.5% of the demographic increase. Consequently, China experienced a net increase of 116.5 million prevalent cases, consistent with its near-zero ASPR decline (EAPC = −0.27) (Table 1).

Kazakhstan achieved a net prevalence decline (−2.26 million) through strong epidemiological improvement (−3.25 million) that outpaced population growth (+0.84 million). Armenia’s net decline (−0.56 million) resulted from epidemiological improvement (−0.54 million) combined with population contraction (−0.09 million). In Uzbekistan and Tajikistan, rapid population growth (+3.73 and +1.47 million, respectively) overwhelmed epidemiological gains (−4.30 and −1.22 million), resulting in net increases (+0.19 and +0.41 million). Turkmenistan presented an intermediate case: despite substantial epidemiological improvement (−0.78 million), demographic pressures from population growth (+0.50 million) and aging (+0.12 million) left only a modest net decline (−0.16 million)-highlighting the delicate balance between competing forces. Kyrgyzstan, Georgia, and Mongolia exhibited smaller net declines (ranging from −0.14 to −0.23 million), driven primarily by epidemiological improvements with relatively limited demographic pressures.

These contrasting trajectories highlight that absolute prevalence trends were profoundly shaped by demographic dynamics, with epidemiological gains alone often insufficient to counterbalance rapid population growth in high-fertility settings.

### 3.3 Health inequality

Across all locations, TB burden was strongly and negatively correlated with SDI in 2023 (ASDR: r = −0.539, p < 0.001; Table 3). However, countries at similar development levels exhibited substantial burden disparities, revealing the influence of health system performance and policy choices (Fig 4). At the low end of the SDI spectrum, Tajikistan (SDI = 0.55, ASDR = 561.7 per 100,000) had the highest burden among all included countries, approximately 1.6-fold higher than Turkmenistan (SDI = 0.68, ASDR = 342.7 per 100,000), despite Turkmenistan’s higher SDI. Mongolia (SDI = 0.66, ASDR = 476.8 per 100,000) also exhibited a substantial burden, though lower than Tajikistan. Among countries with comparable SDI levels, Uzbekistan (SDI = 0.63, ASDR = 174.8 per 100,000) exhibited an ASDR approximately 1.2-fold higher than Kyrgyzstan (SDI = 0.62, ASDR = 150.2 per 100,000)-a disparity that may reflect differences in health system performance rather than development level alone. At the highest development levels, Kazakhstan and China share nearly identical SDI (0.73 and 0.73, respectively), yet Kazakhstan’s ASDR (92.8 per 100,000) is 60% higher than China’s (57.6 per 100,000), highlighting the substantial role of national TB program effectiveness beyond socioeconomic development.

**Table 3:** Health inequality indicators for TB burden in the study region, 1990 vs 2023.

Indicator	Year	Concentration Index (CI) (95% CI)	Relative change	SDI-ASR correlation (r)
**DALYs**	1990	−0.198 (−0.218 to −0.178)	–	–
	2023	−0.076 (−0.089 to −0.063)	↓61.6%	r = −0.539 (*p* < 0.001)
**Mortality**	1990	−0.168 (−0.186 to −0.150)	–	–
	2023	−0.052 (−0.064 to −0.040)	↓69.0%	r = −0.512 (*p* < 0.001)
**Incidence**	1990	−0.185 (−0.202 to −0.168)	–	–
	2023	−0.098 (−0.112 to −0.084)	↓47.0%	r = −0.498 (*p* < 0.001)
**Prevalence**	1990	−0.112 (−0.128 to −0.096)	–	–
	2023	−0.076 (−0.089 to −0.063)	↓32.1%	r = −0.421 (*p* < 0.01)

Correlations were calculated using panel data comprising 10 countries × 34 years (1990–2023), yielding 340 observations per indicator.

**Fig 4:**
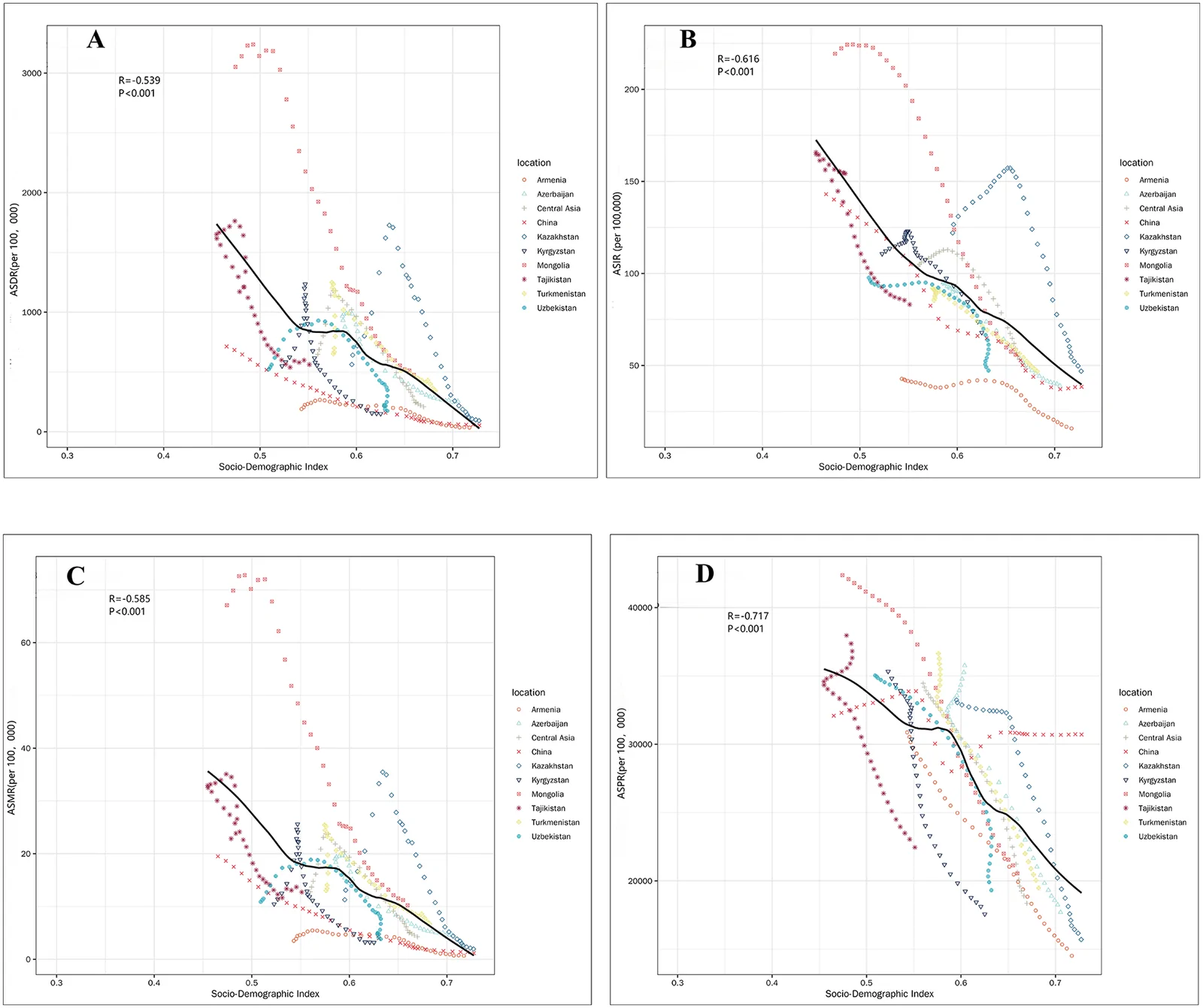
Association between SDI and (A) ASDR, (B) ASIR, © ASMR, (D) ASPR in China and Central Asian countries, 2023. Points represent individual countries; black line = regression slope.

Concentration curves for all four burden indicators lay above the line of equality in both 1990 and 2023, confirming that TB burden has been and remains concentrated among lower-SDI populations (Table 3). However, the curves shifted markedly toward the diagonal over the 34-year period, indicating substantial reductions in socioeconomic inequality across all metrics.

Mortality exhibited the largest decline, with CI improving from −0.168 (95% CI: −0.186 to −0.150) in 1990 to −0.052 (95% CI: −0.064 to −0.040) in 2023-a 69.0% reduction (Table 3). DALYs followed closely, with CI improving from −0.198 (95% CI: −0.218 to −0.178) to −0.076 (95% CI: −0.089 to −0.063), a 61.6% reduction (Table 2). Incidence inequality narrowed by 47.0% (CI: −0.185 to −0.098; Table 3), while prevalence showed the smallest decline at 32.1% (CI: −0.112 to −0.076; Table 3).

Despite this progress, low-SDI populations retained a disproportionate burden in 2023. Tajikistan had the highest absolute CI for ASDR (−346.2), while China had the lowest (−246.4)-a reminder that socioeconomic gradients in TB burden have been compressed, but not eliminated. The SDI-ASDR scatter plots (Fig 4) visualize this compression: the downward shift of the entire curve from 1990 to 2023 confirms that burden reduction has been broadly pro-poor, with the largest absolute gains accruing to the lowest-SDI populations. However, the persistent negative slope reaffirms that low-SDI populations remain at highest risk.

### 3.4 BAPC forecasts (2024–2050)

Prior to forecasting, the predictive performance of the BAPC model using out-of-sample cross-validation (1990–2013 training, 2014–2023 validation) was evaluated. Overall, MAE was 336.81 per 100,000 for China and 2351.63 per 100,000 for Central Asia. By indicator, MAE was lowest for mortality (China: 5.74; Central Asia: 9.59) and highest for prevalence (China: 1052.80; Central Asia: 8801.51) and DALY (China: 240.89; Central Asia: 534.38) (S1 Table). These MAE values were used to construct empirical prediction intervals (2 × MAE) for the projections.

Bayesian age-period-cohort models projected TB burden from 2024–2050 for China and wider Central Asian countries, based on GBD 2023 estimates with quantified uncertainty (Table 4 and Fig 5).

**Table 4:** Projected age-standardized TB burden in China and wider Central Asian region, 2050.

Country	Indicator	2050 projection (mean per 100,000)	SD	95% CI (model‑based)	% reduction 1990–2050
**China**	DALYs	11.6	12.1	(0–35.3)	98.5%
	Mortality	0.08	0.13	(0–0.34)	99.6%
	Incidence	28.6	37.5	(0–102.0)	81.1%
	Prevalence	19,651	18,541	(0–55,950)	40.9%
**Kazakhstan**	DALYs	18.3	22.5	(0–62.4)	96.7%
	Mortality	0.31	0.48	(0–1.25)	97.2%
	Incidence	19.7	12.4	(0–44.0)	83.9%
	Prevalence	10,245	5,678	(0–21,374)	69.2%
**Armenia**	DALYs	32.6	35.7	(0–102.6)	82.9%
	Mortality	0.52	0.76	(0–2.01)	85.1%
	Incidence	22.4	15.2	(0–52.2)	47.6%
	Prevalence	11,877	6,346	(0–24,475)	61.5%
**Azerbaijan**	DALYs	45.8	41.2	(0–126.6)	93.7%
	Mortality	0.89	1.12	(0–3.09)	93.8%
	Incidence	28.5	18.7	(0–65.2)	68.3%
	Prevalence	14,320	5,890	(2,776–25,864)	59.9%
**Georgia**	DALYs	22.4	29.48	(0–79.1)	24.0%
	Mortality	0.18	0.31	(0–0.79)	81.8%
	Incidence	12.8	9.6	(0–31.6)	78.6%
	Prevalence	9,845	4,230	(1,554–18,136)	66.5%
**Kyrgyzstan**	DALYs	95.2	78.6	(0–249.3)	82.7%
	Mortality	1.85	2.12	(0–6.01)	82.3%
	Incidence	42.5	24.8	(0–91.1)	61.6%
	Prevalence	14,230	6,540	(1,412–27,048)	59.7%
**Mongolia**	DALYs	185.6	142.3	(0–464.5)	93.9%
	Mortality	3.85	4.21	(0–12.1)	94.3%
	Incidence	38.4	22.5	(0–82.5)	82.5%
	Prevalence	16,420	7,230	(2,089–30,751)	61.2%
**Tajikistan**	DALYs	128.4	112.3	(0–348.7)	89.0%
	Mortality	2.10	3.20	(0–8.4)	90.8%
	Incidence	38.6	28.9	(0–95.2)	75.2%
	Prevalence	18,923	9,876	(0–38,120)	50.2%
**Turkmenistan**	DALYs	95.7	98.5	(0–288.8)	85.3%
	Mortality	1.80	2.90	(0–7.5)	86.2%
	Incidence	31.2	25.6	(0–81.4)	63.4%
	Prevalence	12,346	4,568	(3,513–21,179)	66.3%
**Uzbekistan**	DALYs	88.4	72.5	(0–230.5)	83.1%
	Mortality	1.72	1.95	(0–5.54)	84.2%
	Incidence	32.5	19.8	(0–71.3)	66.7%
	Prevalence	14,850	5,980	(3,129–26,571)	57.6%
**Central Asia (regional)**	DALYs	43.9	77.7	(0–196.2)	93.3%
	Mortality	0.91	1.84	(0–4.52)	93.2%
	Incidence	16.3	9.35	(0–34.6)	84.8%
	Prevalence	8,703	3,315	(2,206–15,200)	75.5%

**Fig 5:**
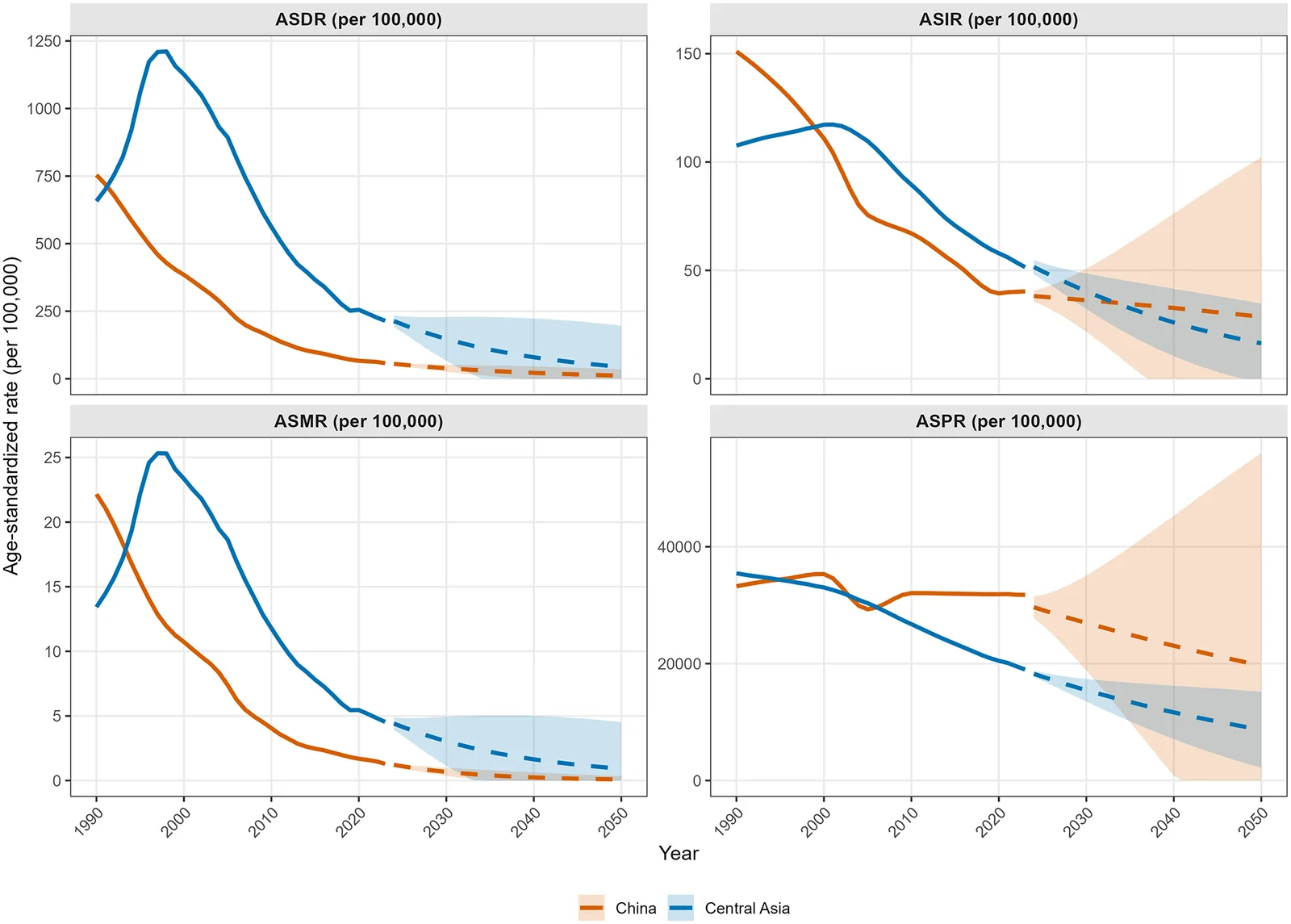
BAPC prediction of age-standardized DALY (ASDR), incidence (ASIR), mortality (ASMR), and prevalence (ASPR) rates in China and the wider Central Asian region, 2024–2050.

By 2050, China’s ASDR is projected to decline to 11.6 per 100,000 (SD = 12.1), a 98.5% reduction from 1990. ASMR is projected to reach 0.08 per 100,000 (SD = 0.13), a 99.6% reduction from 1990, approaching the practical limits of disease elimination. However, Incidence decline will plateau: ASIR declines from 40.3 per 100,000 in 2023 to only 28.6 per 100,000 (SD = 37.5) by 2050, a mere 29% reduction over three decades. Prevalence declines slowly to 19,651 per 100,000 (SD = 18,541), a 40.9% reduction from 1990 (Table 4 and Fig 5).

Central Asian countries show divergent projected trajectories, with uncertainty widening substantially after 2030. Kazakhstan and Armenia, the region’s highest-performing countries, are projected to approach China’s burden levels by 2050. Kazakhstan’s ASDR is forecast to reach 18.3 per 100,000 (SD = 22.5), ASMR to 0.31 per 100,000 (SD = 0.48), and ASIR to 19.7 per 100,000 (SD = 12.4). Armenia’s ASDR is projected to decline to 32.6 per 100,000 (SD = 35.7), with ASIR reaching 22.4 per 100,000 (SD = 15.2). Both countries show moderate uncertainty in their projections. In contrast, Tajikistan and Turkmenistan, the region’s lowest-performing countries, are projected to retain substantial burden with extremely high uncertainty. Such wide prediction intervals are consistent with our model validation strategy, where broader uncertainty bounds were retained for countries with limited routine surveillance data. Tajikistan’s ASDR is forecast to reach 128.4 per 100,000 (SD = 112.3) in 2050, 11 times higher than China’s projected ASDR, with a prediction intervals spanning from near-zero to over 350 per 100,000. ASMR is projected at 2.1 per 100,000 (SD = 3.2), and ASIR at 38.6 per 100,000 (SD = 28.9). Turkmenistan shows similar patterns, with ASDR projected to 95.7 per 100,000 (SD = 98.5) and ASPR to 12,346 per 100,000 (SD = 4,568) (Table 4 and Fig 5). Kyrgyzstan, Uzbekistan, and Mongolia occupy an intermediate position, with projected 2050 ASDR ranging from 95 to 150 per 100,000 and uncertainty intervals that overlap substantially with both higher-and lower-performing countries.

## 4. Discussion

This study provides the first comprehensive, integrated analysis of tuberculosis burden in China and Central Asia from 1990 to 2050, incorporating long-term trends, decomposition of drivers, health inequalities, and Bayesian forecasts. Our findings reveal that China has achieved an advanced epidemiological transition for TB, characterized by near-elimination of mortality and sustained declines in incidence, while Central Asia remains in partial transition with stark internal heterogeneity that population growth is the single greatest threat to reducing absolute TB burden in the region. These results have profound implications for the End TB Strategy and for regional health cooperation under the Belt and Road Initiative [39].

China’s achievements are historically unprecedented for a high-burden country. Between 1990 and 2023, age-standardized mortality declined by 94.2% (EAPC = −8.61), and DALY rates by 92.4% (EAPC = −8.08). By 2050, China’s ASMR is projected to reach 0.08 per 100,000, statistically indistinguishable from zero, and ASDR to 11.6 per 100,000, a 98.5% reduction from 1990. To our knowledge, no country of China’s size and initial burden has ever achieved a comparable mortality reduction, underscoring the exceptional scale of this transition. Decomposition analysis confirms that epidemiological change was the dominant driver, contributing −9.16 million DALYs (142.7% of net reduction) and −268,550 deaths (179.1%), fully offsetting the burden-increasing effects of population growth and aging.

In absolute terms, China’s epidemiological gain in DALY reduction substantially exceeded that of Kazakhstan-the largest contributor in Central Asia-by orders of magnitude, reflecting not only its vastly larger population but also the earlier and more systematic implementation of BCG vaccination and contact investigation [19].

Central Asia achieved substantial per-capita improvements: ASDR declined by 67.4% (EAPC = −4.87), ASMR by 66.2% (EAPC = −4.76), and ASIR by 52.1% (EAPC = −2.52) from 1990 to 2023. Decomposition analysis confirms that epidemiological change was the sole burden-reducing factor, contributing −371,208 DALYs, −6,780 deaths (202.6%), and −45,453 incident cases. However, rapid population growth has substantially diluted these per-capita gains. In incidence, epidemiological changes averted 45,453 cases, but population growth alone added 22,003 cases, offsetting nearly half the epidemiological gain. The net reduction of only 18,285 cases (40% of the raw epidemiological change) demonstrates that demographic drag is the dominant barrier to reducing absolute TB burden in Central Asia. Even if per-capita incidence continues to decline at current rates (−2.5% annually), absolute case numbers will remain essentially flat for decades given projected population growth of 30–40% by 2050 [40].

In this study, decomposition analysis and projections reveal three distinct epidemiological archetypes that require differentiated policy responses:

China has achieved one of the largest sustained reductions in TB mortality among high-burden countries However, Kazakhstan and Armenia are approaching this state, with projected 2050 ASMR of 0.31 and 0.52 per 100,000, respectively. These countries have successfully controlled mortality and are facing an emerging incidence plateau similar to China’s earlier trajectory.

Second, the partial transition archetype characterizes Kyrgyzstan, Uzbekistan, Azerbaijan, Georgia, and Mongolia. These countries have achieved substantial per-capita improvements, but population growth continues to offset epidemiological gains. Their projected 2050 ASDR ranges from 45 to 185 per 100,000, with wide uncertainty intervals reflecting fundamental uncertainty about future decline in the absence of major policy shifts.

Third, countries where demographic growth offset epidemiological improvements are exemplified by Tajikistan-the only country in our study region where absolute deaths, incidence, and prevalence all increased over the 1990–2023 period. In Tajikistan, epidemiological improvements (−772 deaths, −5,592 incident cases, and −1.22 million prevalent cases) were overwhelmed by rapid population growth (+744 deaths, + 5,658 incident cases, and +1.47 million prevalent cases), resulting in net increases of +78 deaths, + 820 incident cases, and +405,388 prevalent cases over the 34-year period. Tajikistan’s TB burden is not declining; it is being masked by denominator growth while absolute case numbers continue to rise. Turkmenistan and Uzbekistan, while not experiencing increases in absolute burden, show similar patterns of demographic overwhelm. Tajikistan represents a high-priority setting, where have not yet achieved TB control; if TB remains uncontrolled in this country, regional elimination is impossible.

Socioeconomic inequality in TB burden declined substantially across all indicators. The Concentration Index for DALYs improved by 61.6% (from −0.198 to-0.076), for mortality by 69.0% (from −0.168 to −0.052), for incidence by 47.0% (from −0.185 to −0.098), and for prevalence by 32.1% (from −0.112 to −0.076). These are major public health achievements, demonstrating that equity-oriented TB control is possible even in highly unequal societies. The 69.0% reduction in mortality inequality, representing the most considerable reduction among all indicators, reflected the broad and equitable expansion of directly observed treatment, short-course (DOTS) and was associated with national TB control policies. This expansion has successfully reached even the most resource-limited settings, aligning with global evidence that TB treatment initiatives can be delivered equitably.

Despite this progress, low-SDI populations remain disproportionately burdened in 2023. Tajikistan had the highest absolute CI for ASDR (−346.2), while China had the lowest (−246.4). The SDI-ASDR scatter plots confirm that the entire curve has shifted downward, burden reduction has been broadly pro-poor, but the persistent negative slope reaffirms that low-SDI populations remain at highest risk. Socioeconomic gradients in TB burden have been compressed, but not eliminated.

The striking SDI-burden mismatch observed in China demonstrates that health system performance and sustained political commitment can decouple TB burden from socioeconomic stage. This offers a realistic model for Central Asian countries at similar or lower SDI levels [10,41].

By 2050, both China and Central Asia are projected to achieve the End TB mortality target under current trends (<1 per 100,000). China’s ASMR of 0.08 per 100,000 and Central Asia’s 0.91 per 100,000 represent 99.6% and 93.2% reductions from 1990, respectively. However, neither region will achieve the End TB incidence target (<10 per 100,000) by 2050 under current trends. China’s projected ASIR of 28.6 per 100,000 and Central Asia’s 16.3 per 100,000 are 2–3 times higher than the target. Critically, Central Asia’s ASIR is projected to be lower than China’s by 2050 (16.3 vs 28.6), a complete reversal of the 1990 ranking. Without large-scale preventive treatment, incidence elimination will remain unattainable this century

The divergent epidemiological trajectories of China and Central Asia demand differentiated policy responses tailored to each region’s stage of transition, with each recommendation directly linked to our data findings.

For China, where decomposition analysis shows that epidemiological change has already achieved near-zero mortality (ASMR 0.08 by 2050) but incidence has plateaued (28.6 per 100,000 by 2050), the paradigm must shift from mortality reduction to prevalence reduction. With near-zero case fatality, the next frontier is shortening the duration of disease. Future national TB strategies may prioritize wider rollout of 4-month rifapentine- moxifloxacin-based regimens, which prior clinical trials demonstrate non-inferior efficacy and improved treatment completion relative to standard 6-month therapy. Critically, with an estimated 350 million latent infections [42,43], Expanded latent TB preventive treatment programs represent a promising intervention to reduce future active TB reactivation risk given China’s large latent infection reservoir. Systematic screening should be expanded to target elderly populations, while health systems must prepare for the aging prevalent cohort, including millions of TB survivors with chronic pulmonary disability who will require integrated TB-chronic disease care for decades [44,45].

For Central Asia, where decomposition analysis reveals that population growth offset nearly half of epidemiological gains in incidence, the overriding priority is accelerating incidence decline to outpace population growth. The current per-capita decline of 2.5% annually is insufficient; a target of 5–6% annual decline is needed to achieve meaningful reductions in absolute burden, consistent with End TB modeling targets. This requires universal access to rapid molecular diagnostics, which currently reaches only 30–60% of the population in most Central Asian countries [5]. Active case finding must be intensified in high-risk populations, including prisons, mining communities, and cross-border migrants along the China-Central Asia economic corridor, where TB incidence has been documented to be substantially higher than national averages. In prisons across Europe and Central Asia, rates can be 5–50 times higher than national averages [46]. Mining communities face elevated risk due to silicosis and overcrowded conditions [47], while migrant workers constitute up to 70% of key population TB cases in countries like Tajikistan [48] and drive cross-border transmission dynamics [49]. Shorter all-oral MDR-TB regimens (9–12 months) should be adopted to improve treatment completion rates, which remain below 70% in several countries [50]. Regional pooled procurement mechanisms could reduce drug costs by 30–50%, accelerating access to novel therapies [51,52]. Empirical evidence from established multi-country procurement programs demonstrates cost reductions of over 50% in the first procurement cycle alone [52], with similar mechanisms achieving 4–82% savings across diverse settings [51,53]. Finally, a cross-border surveillance system is urgently needed to track transmission along the economic corridor and enable coordinated response.

For Tajikistan, the region’s only absolute burden increased where absolute deaths, incidence, and prevalence have all risen, an emergency response is required. Despite per-capita improvements, epidemiological gains have been overwhelmed by explosive population growth. Tajikistan should be designated a regional TB priority country with dedicated funding and technical support.

Regional collaboration offers a strategic pathway to accelerate progress across all countries. The China-Central Asia Tuberculosis Control Research Base, should be fully funded and operationalized as a WHO Collaborating Centre, providing a platform for knowledge transfer, joint research, and policy coordination. China’s experience in DOTS scale-up, universal health coverage, and digital chest X-ray screening is directly relevant to Central Asian contexts. Joint operational research should prioritize shorter regimens in high MDR settings, active case finding in mobile populations, and the intersection of TB with migration, a growing challenge as economic corridors expand regional population movement.

Forecast uncertainty, quantified as the standard deviation of the posterior predictive distribution, increases sharply after 2030 for all indicators and locations, particularly for incidence and prevalence in low-performing countries (Table 4 and Fig 5). By 2050, the prediction intervals for Tajikistan’s ASIR and ASPR span more than an order of magnitude, reflecting fundamental uncertainty about the future pace of decline in the absence of major technological breakthroughs (new vaccines, ultra-short regimens) or policy shifts. These wide intervals should be interpreted not as model failure, but as an honest quantification of the range of possible futures given current data and trends.

The out-of-sample validation confirms that the BAPC model reliably captures long-term declining trends in TB burden. The relatively small MAE values for mortality and incidence indicate that the model performs particularly well for these indicators, which have shown consistent and pronounced declines over the study period. The larger MAE values for DALY and prevalence are consistent with the substantially larger magnitude of these rates and their slower rates of change. The larger overall MAE for Central Asia compared to China reflects the greater heterogeneity in data quality and epidemiological trajectories across the nine countries in the region.

This study has several limitations. First, GBD 2023 estimates rely on modeling in countries with weak vital registration systems; uncertainty intervals for Turkmenistan and Tajikistan are wide, and subnational estimates were unavailable. Second, this study grouped Caucasus nations, Central Asian republics and China into a unified analytical region despite clear geographic separation and heterogeneous socioeconomic and tuberculosis epidemic trajectories across subareas. While we thoroughly justified the inclusion of Caucasus states in this analysis, inherent ecological heterogeneity across these geographically distinct subregions may introduce residual ecological bias and limit causal inference across subregions. Third, forecasts assume no major policy shocks, technological breakthroughs, or emerging threats. Fourth, we did not model HIV co-infection or non-communicable disease comorbidities, which are increasingly important drivers of TB outcomes in aging populations. Moreover, uneven national TB surveillance and vital registration capacity across Central Asian and Caucasian nations creates heterogeneous data reliability, which is reflected in country-specific widths of 95% uncertainty intervals, representing an inherent data quality limitation of the present analysis.

## 5. Conclusion

This study comprehensively analyzed the TB disease burden trends, drivers, inequalities, and future projections in China and the wider Central Asian region from 1990 to 2050. China has accomplished an extraordinary epidemiological transition in TB control, with dramatic declines in age-standardized mortality and DALYs driven by robust epidemiological changes, fully offsetting population growth and aging. Central Asia has achieved notable per-capita TB burden declines but faces stark internal heterogeneity, with rapid population growth overwhelming epidemiological gains in most countries, and Tajikistan even experiencing rising absolute burden. Socioeconomic inequalities in TB burden have significantly lessened across the region, yet low-SDI populations still carry a disproportionate risk. By 2050, the entire region is projected to meet the WHO End TB Strategy’s mortality target, but the incidence target will remain unachieved under current policies. Targeted, differentiated interventions, accelerating incidence decline and cross-border collaboration in Central Asia, and emergency support for high-burden countries like Tajikistan coupled with strengthened China-Central Asia regional cooperation, are critical to advancing regional and global TB elimination.

## Supporting information

S1 Table BAPC model validation: Mean Absolute Error (MAE) and empirical prediction interval half-widths (2 × MAE) by region and indicator. (DOCX)
