# Supplementary material for: Tuberculosis disease burden trends, drivers, and projections to 2050 in China and the wider Central Asian region, 1990–2023
Source: PLoS One. 2026 Oct 9;21(10):e0358349. doi: 10.1371/journal.pone.0358349 (PMC13652273; doi:10.1371/journal.pone.0358349)
Supplement: Supplementary file 1 [file pone.0358349.s001.docx]

Supplementary Table S1. BAPC model validation: Mean Absolute Error (MAE) and empirical prediction interval half-widths (2 × MAE) by region and indicator

| Region | Indicator | MAE (per 100,000) | 2 × MAE (empirical PI half-width) |
| --- | --- | --- | --- |
| **China** | DALY | 240.89 | 481.78 |
|  | Mortality | 5.74 | 11.48 |
|  | Incidence | 47.80 | 95.60 |
|  | Prevalence | 1052.80 | 2105.60 |
|  | **Overall** | **336.81** | **673.62** |
| **Central Asia** | DALY | 534.38 | 1068.76 |
|  | Mortality | 9.59 | 19.18 |
|  | Incidence | 61.02 | 122.04 |
|  | Prevalence | 8801.51 | 17603.02 |
|  | **Overall** | **2351.63** | **4703.26** |

Abbreviations: DALY, disability-adjusted life years; MAE, Mean Absolute Error; PI, prediction interval.

Validation performed using out-of-sample temporal cross-validation: models were fitted on 1990–2013 data and used to predict 2014–2023 rates, which were then compared with observed GBD 2023 estimates. MAE is expressed in the same units as the original rates (per 100,000 population). Empirical prediction interval half-widths are defined as 2 × MAE, used for constructing 95% empirical prediction intervals for the 2024–2050 projections (predicted mean ± 2 × MAE). Central Asia represents the average of nine countries (Kazakhstan, Kyrgyzstan, Tajikistan, Turkmenistan, Uzbekistan, Armenia, Azerbaijan, Georgia, and Mongolia).
